# The Impact of Social Media on Preventive Behavior During the COVID-19 Outbreak in South Korea: The Roles of Social Norms and Self-Efficacy

**DOI:** 10.1177/21582440231184969

**Published:** 2023-07-03

**Authors:** Doo-Hun Choi, Ghee-Young Noh

**Affiliations:** 1 Gachon University, Gyeonggi-do, South Korea; 2 Hallym University, Chuncheon, South Korea

**Keywords:** COVID-19, social media, preventive behavior, social norms, self-efficacy

## Abstract

Social media are important channels to propagate health information and influence preventive behavior during a public health crisis, as witnessed during the coronavirus pandemic (COVID-19). This study explored the association between social media use and preventive behavior during the outbreak of COVID-19 in South Korea. Using the national survey data (*N* = 1,500), the study examined the mediating role of social norms in the association between social media use for news and information about COVID-19 and preventive behavior. In addition, the study tested the moderating effect of self-efficacy on the mediating path of social media use for preventive behavior via social norms. Conducting a moderated mediation analysis method, this study found that social norms mediated the relationship between social media use and preventive behavior (*b* = 0.046). Moreover, the study revealed that the indirect relationship between social media use and preventive behavior through social norms becomes stronger as an individual’s level of self-efficacy decreases (low: *b* = 0.044, middle: *b* = 0.036, and high: *b* = 0.030). The study provides empirical evidence of the beneficial impact of social media use on preventive behavior. The findings of the study recommend promoting messages on social norms through social media for facilitating preventive behavior.

The novel coronavirus disease (COVID-19) has infected more than 500 million people, with over six million death worldwide, resulting in the declaration of a global pandemic by the World Health Organization (2020). South Korea reported over 17 million infections and more than 23 thousand deaths since the first case of COVID-19 in the country was announced in January 2020 (Korea Centers for Disease Control and Prevention [KCDC,], 2020). According to the Centers for Disease Control and Prevention (Centers for Disease Control and Prevention [CDC,], 2020), COVID-19 is transmitted from person to person through respiratory droplets when in close contact, and can cause symptoms such as fever, dry cough, and/or sore throat. Public health experts and government agencies have recommended several specific preventive measures, such as wearing masks, washing hands, and cough etiquette, to prevent the transmission of the disease (Adhikari et al., 2020; CDC, 2020; Omer et al., 2020).

In situations where public health issues such as COVID-19 emerge, it is essential for people to receive timely and appropriate public health information to perceive the threats to public health and successfully prevent transmission (D. H. Choi et al., 2017). Social media, in particular, has played an important role during the previous outbreaks of infectious diseases such as the Middle East Respiratory Syndrome (MERS) and H1N1, in conveying disease-related information and sharing it among users (Jang & Baek, 2019). For example, a survey showed that 71.5% of the respondents used social media primarily to acquire MERS-related information (S. Kim & Yang, 2015). Interestingly, it was observed that people used social media to obtain information relevant to the contagious disease because they thought that they would not receive necessary information from traditional media, such as television and newspapers, during the MERS outbreak in South Korea. (W. Yoo et al., 2016). During the U. S. measles outbreak in 2019, social media were extensively used to express and receive measles-related information and share personal experiences about the infectious disease (S. C. Kim & Hawkins, 2020; Meadows et al., 2019).

As more and more people choose social media (e.g., Twitter or Facebook) to find public health-related information such as news updates and medical information (Seo, 2019; Tang et al., 2018), it is evident that social media use has the potential to facilitate behavioral responses, such as preventive behaviors. Although the existing literature has demonstrated that social media use has a positive relationship with preventive behaviors, such as in the contexts of the 2019 U.S. measles outbreak (S. C. Kim & Hawkins, 2020) and food safety crises in China (Mou & Lin, 2014), the question of how social media use could influence preventive behaviors has not yet been fully explored. To bridge this gap in research, the current study examines the mediating role of social norms in the relationship between social media use and preventive behaviors.

Social norms can affect people’s preventive behavior (Latkin et al., 2013) while social media provide the opportunity to learn social norms (Spartz et al., 2017). Furthermore, to expand our understanding of how social media use and social norms collaboratively impact people’s preventive behaviors, this study investigates the relationship between social media use, social norms, and preventive behaviors and the extent to which this relationship is contingent on an individual’s level of self-efficacy. As prior research showed, self-efficacy indicates individual differences in influencing preventive behaviors (D. H. Choi et al., 2017; W. Yoo et al., 2016). It is, therefore, possible that the impact of social norms on preventive behavior through social media use could differ depending on individual levels of self-efficacy.

Based on this perspective, this study examines the moderated mediation relationships between social media use, social norms, self-efficacy, and preventive behavior using nationwide online survey data collected during the outbreak of COVID-19 in South Korea. Specifically, the study investigates how social media use affects preventive behavior through social norms. Furthermore, this study explores how the mediation path to preventive behavior is influenced by self-efficacy. The results of this study could provide significant implications for risk and health communication by promoting preventive behaviors using social media in the context of an infectious disease outbreak. Figure 1 illustrates the proposed associations among the variables in this study.

**Figure 1.**
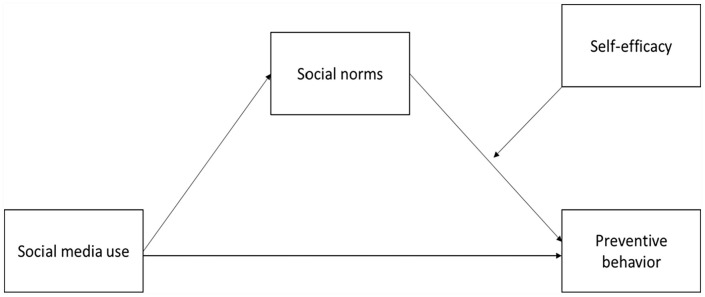
Proposed research model.

## Social Media Use and Preventive Behavior

When a public health crisis such as COVID-19 occurs, one of the most important public responses is practicing preventive behaviors. Preventive behaviors represent any behaviors undertaken to avoid or decrease risks from diseases or negative health outcomes (D. H. Choi et al., 2018). In general, people participate in preventive behaviors, such as getting vaccinated, undergoing mammography tests, and using contraceptives to inhibit serious future problems and sustain their health (D. H. Choi et al., 2018). In particular, in the case of an infectious disease outbreak, such as MERS or COVID-19, public health experts and government agencies recommend several specific preventive measures, such as wearing masks, washing one’s hand, and covering one’s face when coughing or sneezing with a tissue or cloth, to avoid transmission of the disease (CDC, 2020; J. S. Choi & Kim, 2016). The public should, therefore, engage in preventive behaviors during the outbreak of an infectious disease, especially until pharmaceutical interventions such as vaccines are accessible.

The use of social media for accessing health-related news and information can impact an individual’s preventive behavior. Since people would like to be informed about the preventive measures to be taken, health and risk communications need to offer them accurate information on how to respond to the public health emergency, thus promoting appropriate behavioral responses (Verroen et al., 2013). Social media can help people in obtaining health or disease-related news and information, such as safety instructions or quarantine restrictions, and exchanging the information with existing online social contacts, such as friends and family members, in real-time, which can motivate people to engage in preventive behaviors (Oh et al., 2021). For example, during the 2015 MERS outbreak in South Korea, a survey found that social media use increases preventive behaviors, such as wearing masks and handwashing (Seo, 2019). More recently, a survey conducted in China found that social media use was positively associated with preventive behaviors, including handwashing and following coughing etiquette during the COVID-19 pandemic (Li & Liu, 2020). On the other hand, another study argues that social media use can decrease preventive behavior during a pandemic (e.g., Allington et al., 2021). These diverging views raise the following research question:

RQ1: How is social media use associated with preventive behaviors?

## Mediating Role of Social Norms in Preventive Behaviors

Social norms refer to codes of conduct that informally govern members’ behavior in groups and societies (Rimal & Real, 2003). Generally, people perceive social norms as the thoughts, feelings, or behaviors that would typically be demonstrated by close others, such as family members, friends, or coworkers in a given situation (Turner, 1991). Specifically, social norms include perceptions regarding what ought to be done in a certain way (i.e., injunctive norms; e.g., my family thinks that I should engage in preventive behaviors) and perceptions of what is commonly done by others in a specific situation (i.e., descriptive norms; e.g., how widespread practicing preventive behaviors is among close ones; Carpenter & Amaravadi, 2019; Cialdini et al., 1990). Social norms play a significant role in helping people understand which behaviors would be desirable or normative in a particular circumstance (Lombardi et al., 2019).

More importantly, it has been demonstrated that social norms can affect people’s behavioral change in the area of public health (e.g., Lapinski et al., 2013; Schoenaker et al., 2018). The notion of social norms suggests that people’s behaviors are largely influenced by perceptions of how other individuals in one’s social group behave (Perkins & Berkowitz, 1986; Phua, 2013). As an individual scans their social environment using mediated sources such as direct interpersonal contact or social media, it can lead to them making inferences about the behavior of others, which they use to determine their own behavior (Spartz et al., 2017). While some studies point out that social norms can play a role in provoking risky behaviors, such as drinking and driving among young drivers (e.g., Geber et al., 2021) and illicit drug use among college students (e.g., Kollath-Cattano et al., 2020), other scholars in the field of health communication have suggested the beneficial role of social norms in promoting healthy behaviors. For example, a survey study showed that perceived support for human papilloma virus (HPV) vaccination from friends and parents helped young women to decide to get the vaccine (Visser et al., 2011). A multinational study conducted in China, Singapore, and Canada also found that social norms are positively associated with SARS-preventive behaviors, such as wearing facemasks or washing hands with soap (Cheng & Ng, 2006).In particular, when facing uncertainty in a social situation, such as that of COVID-19, social norms provide context-appropriate cues for behaving appropriately (Spartz et al., 2017). Additionally, social norms can serve as a form of social pressure that causes people to engage in specific behaviors (Wombacher et al., 2017), particularly for protecting themselves from risks, by taking actions such as wearing masks (e.g., Duong et al., 2021; Santana et al., 2020). Based on these observations, the current study states that social norms toward preventive behavior during the outbreak of COVID-19 can play an important role in promoting preventive behavior among people.

Social media use can have an impact on the formation of perceived social norms. Social environments offer meaningful cues regarding how individuals should behave (Yanovitzky & Stryker, 2001). For example, as an important aspect of the current social environment, mass media (e.g., television and newspapers) play a significant role in helping people to perceive social norms (Chia & Gunther, 2006; Lapinski & Rimal, 2005). Exposure to media content portraying diverse behaviors can shape perceptions of the majority of the behavior (Wombacher et al., 2017). For instance, a survey found that media use for obtaining information about condom use is associated with perceptions of social norms regarding safe sex behavior (Hong & Kim, 2020). In recent years, as numerous people have opted for social media for the consumption of news and information content, it has the potential to influence perceptions of social norms about a particular behavior (S. Kim et al., 2015).

Social norms are formed, spread, and reinforced through communication among people (Wombacher et al., 2017). In particular, as people communicate and interact with important others, such as family members, friends, and coworkers, they have the opportunity to discern which behavior is socially acceptable and expected (Yanovitzky & Stryker, 2001). Social media can facilitate such interaction and communication through online social networks, allowing users to adopt behaviors that are approved by significant others. This suggests that social media are an effective means for making actions visible to other users and provide an opportunity to learn a specific behavior from friends and peers (Hynes & Wilson, 2016). In particular, social media provide social media metrics (e.g., the number of shares, likes, and comments) displayed alongside content, such as news articles or news blogs. This information helps infer what other users think about the content (Lee & Su, 2020; Spartz et al., 2017). Moreover, social media metrics can enable users to believe what is approved by other users and implement the actions recommended (e.g., what ought to be done) in the content (Lee-Won et al., 2016), which may function as a form of social pressure, informing them how they should behave.

Based on the above discussion, this study argues that social media use for the consumption of news and information on COVID-19, including preventive behavior methods, can impact the formation of perceptions of social norms about preventive behavior. More specifically, this study suggests that social norms play a mediating role in the association between social media use and preventive behavior. Social media use can help shape social norms, which may, in turn, facilitate preventive behavior. Thus, the following hypothesis is proposed:

H1: Social media use is positively associated with social norms, which may have a positive relationship with preventive behavior.

## Moderating Role of Self-Efficacy

Self-efficacy refers to an individual’s belief in their own ability to perform a particular behavior successfully (Bandura, 1997). For example, in the context of health, people may believe that they can control chain smoking or habitual drinking. In particular, self-efficacy helps individuals to handle certain difficult tasks they are facing (D. H. Choi et al., 2017). When public health emergencies such as COVID-19 happen, self-efficacy can play a major role in encouraging people to overcome social difficulties (Bandura, 1997; D. H. Choi et al., 2017). In addition, since self-efficacy results in favorable outcomes, people with high self-efficacy anticipate these results and behave accordingly (Bandura, 2004). More importantly, it has been suggested that self-efficacy has the potential to promote healthy behavior and contribute to disease prevention (Grembowski et al., 1993; S. W. Yoo et al., 2018). The extended parallel process model (EPPM) posits that self-efficacy can influence engaging in health behaviors, such as preventive behaviors (e.g., Jang & Park, 2018; W. Yoo et al., 2016). According to the EPPM, self-efficacy helps people estimate their ability to conduct the recommended behavior to avert a health risk or threat. Compared to people with lower levels of self-efficacy, those with higher levels of self-efficacy are more likely to practice healthy behaviors, such as taking preventive measures during the H1N1 pandemic, doing aerobic exercise, or following a sensible diet, (Bults et al., 2011; Grembowski et al., 1993; Ievers-Landis et al., 2003). Thus, it is likely that individuals with high levels of self-efficacy would engage in preventive behaviors to protect themselves against COVID-19.

More interestingly, in addition to testing the mediating mechanism of social media use on preventive behavior through social norms, the current study also examines a moderated mediation mechanism by suggesting that individual differences, such as self-efficacy, moderate the mediating association between social media use and preventive behavior through social norms. As self-efficacy indicates individual differences in the extent to which a person believes that they have the capability to control a situation, the impact of social norms on preventive behavior could differ according to individual levels of self-efficacy. Therefore, investigating the moderating role of self-efficacy could indicate the conditions under which the mediating association would differ (Y. Kim & Chen, 2015). For example, prior research has suggested that self-efficacy can play a moderating role in the relationship between media messages and preventive behavior, such as skin cancer self-examination (van ’t Riet et al., 2010). It is plausible that since people have different levels of self-efficacy, their perceptions of social norms regarding preventive behaviors, obtained from social media use about COVID-19, could produce different levels of preventive behaviors. Given this consideration, examining the moderating effect of self-efficacy in the mediated association between social media and social norms on preventive behavior represents an important perspective in this area of research. Examining self-efficacy as a moderator will provide a better understanding of the role of individual differences in increasing or weakening the impact of social media use and social norms on preventive behavior. Because little research has been conducted on the moderated mediation relationship among social media use, social norms, and self-efficacy in preventive behavior, a directional hypothesis about the relationship could not be addressed. Thus, this study proposes the following research question.

RQ2: How does self-efficacy moderate the impact of social media use on preventive behavior through social norms?

## Methods

### Data^1^

The data used for this study were collected from a national online panel survey of South Korean adults conducted by Global Research, a major research firm in South Korea, over 1 week from February 24 to March 3, 2020. The survey period coincided with one of the significant peaks of the COVID-19 outbreak in South Korea. The survey company secured an online panel of over one million people with national representation on the basis of age, gender, and region using a proportionate quota sampling approach. Among the entire pool, 127,362 panel members, who were randomly chosen via a computer algorithm, received the survey invitation email to participate in the current study. Out of the total emails sent, 2,225 individuals responded to the survey participation, and 725 cases out of these were excluded due to inadequate or incomplete answers in the questionnaire. As a result, 1,500 people finished the survey, producing a completion rate of 67.4% and all 1,500 responses were included in the analysis. This research was approved by the Institutional Review Board of Hallym University, South Korea (IRB no. HIRB-2020-015).

### Measures

#### Social Media Use

The study measured *social media use* by using a single item on a 5-point scale ranging from “1 = never” to “5 = very often,” when asked how often the respondents had seen or heard news and information in relation to preventive methods about COVID-19 on social media, such as Instagram, Facebook, and Twitter during the past month (*M* = 3.61, *SD* = 1.14).

#### Social Norms

Guided by Fishbein and Ajzen (2011)’s recommendation, injunctive and descriptive norms were combined as a measure of social norm. *Social norms* were measured using a 5-point scale ranging from “1 = strongly disagree” to “5 = strongly agree” by asking respondents to rate their level of agreement with the following four statements regarding preventive behavior: (1) “Most people who are important to me (e.g., family members and friends) think that I should practice COVID-19 preventive behavior,” (2) “Most people who are important to me expect that I will engage in COVID-19 preventive behavior,” (3) “Most people who are important to me want that I practice COVID-19 preventive behavior,” and (4) “Most people who are important to me engage in COVID-19 preventive behavior.” These responses were averaged together to construct an index of social norms (Cronbach’s alpha = .82, *M* = 4.28, *SD* = 0.59).

#### Self-efficacy

*Self-efficacy* regarding COVID-19 was measured using three items on a 5-point scale ranging from “1 = strongly disagree” to “5 = strongly agree” by asking respondents how much they agree with the following statements: (1) “I can avoid COVID-19,” (2) “I can recover even if I was infected from COVID-19,” and (3) “I know how to avoid COVID-19.” The three items were averaged together to create an index of self-efficacy (Cronbach’s alpha = .74, *M* = 3.65, *SD* = 0.75). The measurement items were adopted and modified from previous studies (e.g., Han et al., 2014; W. Yoo et al., 2016).

#### Preventive Behavior

*Preventive behavior* was assessed on a 5-point scale ranging from “1 = not at all” to “5 = very much” by asking how frequently respondents engaged in the following preventive behaviors at that time: (1) “I wear masks when I go out,” (2) “I wash my hands frequently,” (3) “I do not touch my eyes, nose, and mouth with unwashed hands,” and (4) “I comply with recommended cough etiquette, such as covering my mouth and nose with a sleeve or a tissue when I cough or sneeze.” Responses to these four items were averaged to construct an index of preventive behavior (Cronbach’s alpha = .75, *M* = 4.39, *SD* = 0.54).

#### Control Variables

The study included the variables of age, gender, education, income, political ideology, and history of respiratory diseases in the analysis for controlling the potential confounding effects of the hypothesized relationships. The mean age of the sample was 40.31 (*SD* = 10.91), 48.7% of the respondents were female; the median of the participants’ education level was college graduate, and the median monthly household income level was KRW 4.00 to 5.00 million (approx. USD 4,000 to 5,000). Of the sample, 90.1% reported that they had not experienced any respiratory diseases in the previous year. Political ideology was measured using a 5-point scale ranging from “1 = very liberal” to “5 = very conservative” (*M* = 2.82, *SD* = 0.75).

Table 1 presents the demographic characteristics of the sample.

**Table 1.** Socio-Demographic Profile.

	Participants (%)
Age in years	
19–29	23.7
30–39	22.3
40–49	26.5
50 over	27.5
Gender	
Male	51.3
Female	48.7
Education	
Less than high school graduate	21.6
College degree	69.4
Graduate degree or higher	9.1
Income (monthly household income)	
Less than ₩ 2,000,000	11.9
₩ 2,000,000–₩3,999,999	31.8
₩ 4,000,000–₩6,999,999	32.0
₩ 7,000,000 or more	14.2

### Analysis

To investigate the hypothesized relationships in the suggested model, a moderated mediation analysis was performed using the SPSS PROCESS macro (Hayes, 2018). The PROCESS macro simultaneously enables testing of the direct effects between variables based on ordinary least square regression and examining the indirect effects with a bootstrapping method (Hayes, 2018). This study employs the bias-corrected bootstrap method with 5,000 samples and a 95% confidence interval (CI). Specifically, we use the PROCESS Model 4 was used to examine the mediating relationship between social media use and preventive behavior through social norms. Furthermore, the study employs the PROCESS Model 14 to estimate whether the impact of social media use on preventive behavior through social norms is contingent on self-efficacy (i.e., the moderated mediation model).

## Results

**Figure 2.**
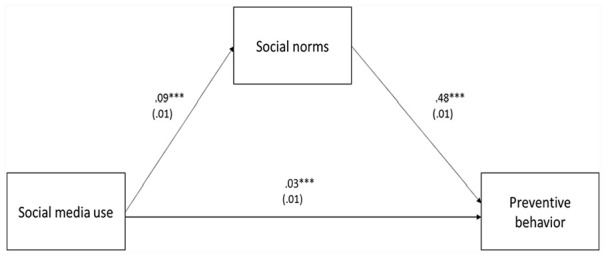
Mediation model of social media use, social norms, and preventive behavior.

The present study used the PROCESS macro Model 4, which produces both the direct relationship (see Figure 2) and the indirect effect among the variables (see Table 1). Figure 2 shows the unstandardized path coefficients and statistical significance in the direct associations among social media use, social norms, and preventive behavior. The study results demonstrated a positive relationship between social media use and social norms (*b* = 0.09, *SE* = 0.01, *p* < .001). In response to RQ1, social media use had a positive association with preventive behavior (*b* = 0.03, *SE* = 0.01, *p* < .001). Moreover, the results indicate that social norms have a positive relationship with preventive behavior (*b* = 0.48, *SE* = 0.01, *p* < .001).

As presented in Table 2, the results point to the indirect impact of social media use on preventive behavior through social norms (*b* = 0.046, *SE* = 0.007, 95% CI [0.032, 0.061]). Thus, the result supports H1.

**Table 2.** Indirect Effect of Social Media Use on Preventive Behavior Through Social Norms.

Indirect Path	Estimate	*SE*	CI
Social media use → Social norms → Preventive behavior	0.046	0.007	0.032–0.061

*Note*. Unstandardized regression coefficients and corresponding standard errors are reported; CIs are bias-corrected 95% confidence intervals for the indirect effects (Bootstrap *N* = 5,000).

**Figure 3.**
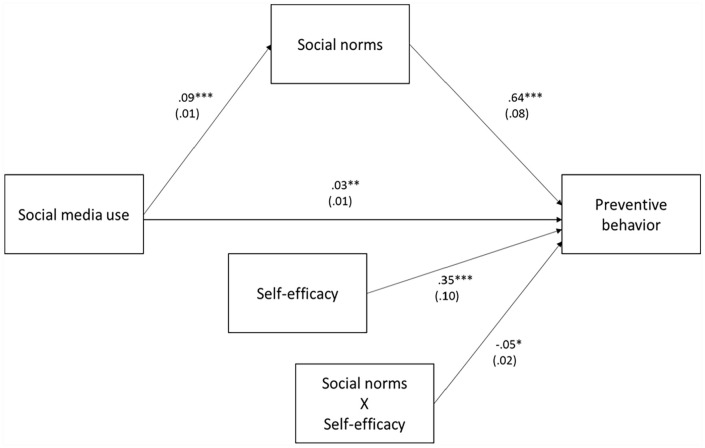
Moderated mediation model of social media use, social norms, self-efficacy and preventive behavior.

Additionally, we ran the PROCESS macro Model 14 was used to find the moderated mediation effect, yielding direct effects (see Figure 3) and the moderated mediated effect (see Table 2). Figure 3 illustrates the direct effect that social media use, social norms, self-efficacy, and the interaction term (social norms ’ self-efficacy) were entered in predicting preventive behavior. Social media use had a positive relationship with social norms (*b* = 0.09, *SE* = 0.01, *p* < .001) and preventive behavior, (*b* = 0.03, *SE* = 0.01, *p* < .01), respectively. In addition, social norms were found to be positively associated with preventive behavior (*b* = 0.72, *SE* = 0.08, *p* < .001). Furthermore, the data analysis found that self-efficacy was positively related to preventive behavior (*b* = 0.50, *SE* = 0.10, *p* < .001). The interaction effect of social norms and self-efficacy on preventive behavior was statistically significant (*b* = −0.08, *SE* = 0.02, *p* < .001).

In response to RQ2, the results of the moderated mediation analysis demonstrate that there are significant conditional indirect effects of social media use on preventive behavior. Table 3 presents the different degrees of indirect effects of social media use on preventive behavior through social norms according to the level of self-efficacy. Specifically, the indirect effect of social media use on preventive behavior through social norms was significant for low (*b* = 0.044, *SE* = 0.007, 95% CI [0.030, 0.059]), middle (*b* = 0.036, *SE* = 0.006, 95% CI [0.024, 0.049]), and high (*b* = 0.030, *SE* = 0.006, 95% CI [0.019, 0.044]) levels of self-efficacy. The results indicate that the indirect effect of social media use on preventive behavior through social norms becomes stronger as the level of self-efficacy decreases.

**Table 3.** Moderated Mediation Model: Conditional Indirect Effects of Social Media Use on Preventive Behavior Through Social Norms Moderated by Levels of Self-efficacy.

Mediator	Moderator Condition	Estimate	SE	CI
Social norms	Low	0.044	0.007	0.030–0.059
Social norms	Middle	0.040	0.006	0.028–0.054
Social norms	High	0.037	0.006	0.025–0.051

*Note*. Unstandardized regression coefficients and corresponding standard errors are reported; Conditions for moderator (self-efficacy) are the mean and one standard deviation below and above from the mean. CIs are bias-corrected 95% confidence intervals for the indirect effects (Bootstrap *N* = 5,000).

## Discussion

The present study explored how social media use is associated with preventive behavior through social norms as well as how the mediating relationship differs depending on an individual’s level of self-efficacy, by analyzing data from a national online panel survey of South Koreans for a week during the COVID-19 outbreak. Since social media play an important role in propagating health information during a public health crisis, this study argues that social media use can help people to engage in preventive behavior to protect themselves from the outbreak of an infectious disease. The findings of the current study advance the understanding of the impact of social media on preventive behavior, particularly in promoting preventive behavior for individuals with a lower level of self-efficacy. This study suggests that social media can assist in the development of beneficial health and risk communication strategies for the public in the case of future public health crises.

More specifically, this study identified an indirect relationship model, wherein social norms mediate the association between social media use and preventive behavior. This finding suggests that individuals who use social media more frequently for news and information about COVID-19 are more likely to perceive preventive behavior practices as social norms, which may, in turn, be positively related to engaging in preventive behavior. Therefore, social media could serve as an important source of social norms, helping people perceive normative cues about preventive behavior shown by other users through online networks (Spartz et al., 2017; Yang & Wu, 2021). This observation is positively related to practicing frequent preventive behavior against the infectious diseases, such as COVID-19. That is, people who perceived the performance and approval of preventive behavior against COVID-19 were more likely to engage in preventive behaviors. This perception encourages them to comply with the behaviors of the majority in their social group (Bandura, 2004). In particular, during the infectious disease outbreaks, social norms may function as a form of social pressure by encouraging people to engage in preventive behavior, and social media play a significant role in helping people to perceive such social norms related to preventive behavior. The findings of this study support the proposition that the significant association between social media use and preventive behavior is maintained in the context of social norms. This suggests that social media use has a positive relationship with preventive behavior, particularly by providing important normative signals about the preventive behavior of people close to them to protect themselves against the infectious disease.

Furthermore, the study results indicated that the indirect path to preventive behavior differed depending on an individual’s level of self-efficacy. This finding supports the notion that the impact of media use on behavioral outcome variables can differ depending on individual differences (e.g., Y. Kim & Chen, 2015). From a theoretical perspective, this result advances that social norms function as a significant mediating mechanism, however, this mediating relationship with preventive behavior is contingent on individuals’ self-efficacy, indicating that self-efficacy significantly moderates the mediating paths. The relationship between social media use and preventive behavior through social norms was higher for individuals with a lower level of self-efficacy than for those with a higher level of self-efficacy. Specifically, compared to individuals with a higher level of self-efficacy regarding COVID-19, those with a lower level of self-efficacy regarding COVID-19 could benefit more from social media, in this respect, despite the main effect of self-efficacy being that respondents with a higher level of self-efficacy engaged more frequently in preventive behavior than those with a lower level of self-efficacy. When people with a lower level of self-efficacy for COVID-19 use social media often for news and information about the corona virus, they are more likely to obtain greater benefits from perceiving social norms on preventive behavior than those with a higher level of self-efficacy, who already perceive social norms that are positively associated with preventive behavior practice. It is possible that individuals with low self-efficacy would use social norms as a promoter to engage in preventive behaviors compared to those with high self-efficacy. This finding may suggest that social media would be a beneficial tool, particularly for people with low self-efficacy for COVID-19, in perceiving social norms of preventive behavior and helping them to practice more preventive behavior.

## Limitations and Suggestions for Future Research

Although the present study reveals important findings, it has some limitations. First, this study is based on cross-sectional survey data, and hence, it is difficult to justify the causal claims of the associations observed. In other words, the study results cannot build temporal order nor eliminate the possibility of reverse causality of the relationships between social media use, social norms, and preventive behavior. Nevertheless, as discussed above, the present research model was suggested based on theoretical grounds, strong inferences, and empirical evidence derived from previous studies. Although the current interpretation of the findings is congruent with prior literature, future research needs to adopt a longitudinal approach to establish stronger causal arguments.

Second, the study used self-reported preventive behavior data rather than directly measuring actual actions. Even though self-reported behavior measures have been broadly employed in research, a self-reported measurement may be limited because it is vulnerable to social desirability bias. Nevertheless, the survey approach adopted in this study may be suitable for further exploring the association between social media use and preventive behavior, which could offer more generalizable nationwide results.

Third, the social media use measure used in the study relied on respondents’ retrospective recall of exposure to news and information about COVID-19 on social media. It is possible that respondents’ memory could be biased and inaccurate in capturing their behavior (Prior, 2009; W. Yoo et al., 2020). Future studies should use real-time behavioral data to gauge social media use. Additionally, another shortcoming regarding the social media use measure is that this study used a single item to assess social media use, which may raise a concern about reliability and validity. However, some prior studies also have employed a single item to investigate the impact of social media as an information source in the contexts of health and risk communication (e.g., Oh et al., 2021; W. Yoo et al., 2020). Nevertheless, future research needs to use more items to capture complete social media use, which may yield robust outcomes.

Fourth, this study did not distinguish between injunctive norms and descriptive norms when examining the effects of social norms on preventive behavior, regardless of the fact that injunctive and descriptive norms can be tested with a composite norm variable (Fishbein & Ajzen, 2011; M. Yzer, 2012). Even though both injunctive norms and descriptive norms can foster one’s behavior by offering information about what represents desirable behavior in a given context (Lapinski & Rimal, 2005), some studies have also suggested that the two types of norms may play different roles in impacting health-related behaviors (e.g., Ho et al., 2014; Smith-McLallen & Fishbein, 2008; Stok et al., 2014). Future research may need to explore the distinctive roles of both injunctive and descriptive norms on preventive behaviors in the context of COVID-19 by including more items on such norms.

In future research, it would be an interesting topic to examine how social media use affects people’s COVID-19 vaccine uptake through social norms. Such research would be important for understanding how to promote COVID-10 vaccination as a preventive behavior. Exploring the associations among social media use, social norms, and self-efficacy could offer key implications for facilitating vaccination in future infectious disease outbreaks.

In addition, future research also needs to consider the dynamics of each community, such as suburban or urban, regarding the impact of social media on preventive behavior. If the epidemic continues, tailored strategies for each community in the area of public health and health communication should be developed separately for responding to the risk of the infectious disease.

Finally, future research should consider the importance of demographic factors, such as age and gender, in affecting preventive behavior. For example, it would be interesting to examine whether the younger age group or older age group obtain a stronger benefit from social media use to engage in preventive behavior. Future research may contribute to understanding the differential role of demographic factors. In this regard, exploring the demographic factors may help establish tailored recommendations for influencing preventive behavior.

## Conclusion

With these considerations in mind, this study contributes to a better understanding of the mechanism underlying the relationship between social media use and preventive behavior, particularly focusing on the mediating role of social norms and the moderating role of self-efficacy. Consistent with the findings of previous studies that social media use has significant improvement in health behavior change (Maher et al., 2014), this study provides empirical evidence of the benefits of social media use on preventive behavior for COVID-19, especially being greater for individuals with lower self-efficacy. This study elucidates the significant role of social norms derived from social media use through the provision of a positive pathway to preventive behavior. The study results have critical implications given the concerns that some people are reluctant to engage in preventive behavior during the outbreak of a contagious disease.

In addition, the findings of the current study have key practical implications for public health, underscoring the importance of preventive behavior, during the outbreak of a contagious disease. Given the new media environment, where social media serve as an important channel for disseminating health and risk information to people, public health communication practitioners and public health policymakers need to leverage the role of social media for health communication intervention in the event of future emergencies. Specifically, when developing public campaigns to promote preventive behavior, public health communicators need to design social norms messages that portray commonly accepted behavior by other people through social media. In particular, public health communication experts and public health policymakers need to utilize the beneficial role of social media by targeting specific segments of the general public, particularly for those with a lower level of self-efficacy for COVID-19. Effective health communication should help the public to engage in appropriate preventive behavior to avoid the spread of an infectious disease.

## Appendix A

**Table:**

Construct	Measurement items
Social norms Preventive behavior	1. Most people who are important to me (e.g., family members and friends) think that I should practice COVID-19 preventive behavior. 2. Most people who are important to me expect that I will engage in COVID-19 preventive behavior. 3. Most people who are important to me want that I practice COVID-19 preventive behavior. 4. Most people who are important to me engage in COVID-19 preventive behavior.
Self-efficacy	1. I can avoid COVID-19. 2. I can recover even if I was infected from COVID-19. 3. I know how to avoid COVID-19.
Preventive behavior	1. I wear masks when I go out. 2. I wash my hands frequently. 3. I do not touch my eyes, nose, and mouth with unwashed hands. 4. I comply with recommended cough etiquette, such as covering my mouth and nose with a sleeve or a tissue when I cough or sneeze.

## Notes

1. The data that support the findings of this study are available from the corresponding author upon reasonable request.
